# Subjective well-being, drug attitude, and changes in symptomatology in chronic schizophrenia patients starting treatment with new-generation antipsychotic medication

**DOI:** 10.1186/s12888-018-1791-y

**Published:** 2018-06-28

**Authors:** Christian G. Widschwendter, Georg Kemmler, Maria A. Rettenbacher, Nursen Yalcin-Siedentopf, Alex Hofer

**Affiliations:** Medical University Innsbruck, Department of Psychiatry,Psychotherapy, and Psychosomatics. Division of Psychiatry I, Anichstrasse 35, 6020 Innsbruck, Austria

## Abstract

**Background:**

Non-adherence to medication remains a major challenge in the long-term management of patients with schizophrenia. Next to lack of insight into the illness, adverse effects of antipsychotic drugs, cognitive deficits, poor therapeutic alliance, reduced quality of life, missing social support, and negative attitudes toward medication are predictors of non-adherence. This study examined potential correlations between attitudes toward antipsychotic drug therapy, subjective well-being, and symptom change in patients with chronic schizophrenia.

**Methods:**

30 patients with schizophrenia starting monotherapy with a new-generation antipsychotic were included into the study. The Drug Attitude Inventory (DAI) and the Subjective Well-being under Neuroleptic Treatment Scale, short form (SWN-K), were administered after 2, 4, and 12 weeks of treatment. At the same points in time and at baseline, psychopathological symptoms were rated by means of the Positive and Negative Syndrome Scale (PANSS), and functioning was assessed by means of the Global Assessment of Functioning Scale (GAF). Antipsychotic induced side effects were evaluated by using the Udvalg for Kliniske Undersogelser (UKU) Side Effect Rating Scale.

**Results:**

Study participants had a mean age of 37.5 ± 9.7 years, baseline symptoms were mild. The PANSS total score improved significantly from baseline to weeks 4 (*p* = .003) and 12 (*p* = .001), respectively. Neither the DAI total score nor the SWN-K total score changed significantly over the course of time. The severity of symptoms was not correlated with drug attitude at any time point but was negatively correlated with wellbeing at weeks 2 (*r* = −.419, *p* = .021) and 4 (*r* = −.441, *p* = .015). There was no significant correlation between DAI and SWN-K total scores at any time point.

**Conclusions:**

Next to showing that the DAI and the SWN-K measure different aspects of subjective experiences during antipsychotic treatment these findings emphasize the use of both instruments to optimize adherence to medication.

## Background

Despite advances in the pharmacotherapy of schizophrenia poor-adherence to antipsychotic medication remains a major challenge in the long-term treatment of patients. Most studies reported on high frequencies of partial and non-adherence in this patient group with different factors being relevant in this context [1]: next to lack of insight into the illness negative attitudes towards the illness and the medication, negative past experiences with the illness and its treatment, and the lack of support systems are known to be strong predictors of non-adherence [2–5]. This is of particular relevance, since non-adherence is one of the most important risk factors for relapse, rehospitalization, and treatment resistance as well as for substance abuse, violence, arrests, suicide attempts, and impaired long-term functioning [6]. During the era of first generation antipsychotics, the emphasis of treatment lay on objective outcome parameters, e.g., a reduction of positive symptoms, and little research was done with regard to the patients’ subjective perspective [7]. With the introduction of new generation antipsychotics treatment goals became more ambitious and more attention was paid to patients’ complaints, e.g., cognitive slowing, affective blunting or loss of spontaneity and volition [8, 9]. Next to that, the interest in the patients’ view of quality of life has significantly increased over the last decades [10].

Most patients with schizophrenia, if not acutely psychotic or experiencing severe cognitive impairments, are able to complete self-rating scales in a meaningful way, and it has been shown that patients’ and psychiatrists’ perspectives on antipsychotic treatment differ considerably [11–13]. Several scales have been developed to assess quality of life in patients with schizophrenia [7] and both the impact of pharmacological therapy on quality of life and the relationship between subjective experience and attitudes and adherence to medication attracted attention over the past decades. In 1983, Hogan et al. developed the Drug Attitude Inventory (DAI) with the objective to quantify patient’s subjective experience of treatment with antipsychotic drugs [14]. Importantly, positive attitudes towards medication have been associated with lower symptom levels, and treatment response has been demonstrated to be positively correlated with a positive drug attitude [3, 15–19]. Later, Naber et al. developed the “Subjective Well-being under Neuroleptic Treatment” scale (SWN), which captures the subjective experiences of patients during antipsychotic drug treatment [12, 20] and has inconsistently been shown to correlate with current symptomatology or changes in psychopathology [20–22].

Although some authors claimed that both instruments examine quality of life [7, 23], merely the SWN is highly correlated with this subjective outcome variable [12]. Initially designed to predict drug compliance, the DAI focuses on the subjective effects of antipsychotic medication and on values and attitudes toward the illness and health without differentiating between these issues [24]. The SWN, on the other hand, measures quality of life rather than experiences attributed to antipsychotic medication without distinguishing between pharmacogenic or morbogenic components [7]. However, consensus on a uniform definition of the multidimensional and partially subjective concept of quality of life [25–27] is still missing. In contrast to “overall quality of life”, the term “health-related quality of life” is used in the context of medical treatment and research and includes three major determinants: subjective well-being, functioning in daily life, and external resources. Consequently and in contrast to the concept of “well-being”, which can be seen as a psychological and emotional state, the concept of quality of life also encompasses physical, social, cognitive and functional aspects [24, 28].

The aim of this study was to assess the associations between subjective well-being and attitudes towards antipsychotic medication as well as the interrelations between these issues and both symptoms and functioning in patients with chronic schizophrenia starting monotherapy with a new-generation antipsychotic drug.

## Methods

### Subjects and experimental design

Patients aged between 18 and 65 years and starting treatment with a new-generation antipsychotic drug were included into a prospective longitudinal study to build a drug monitoring register. Subjects were recruited via a specialized outpatient service for patients with psychotic disorders or they were inpatients. They met the diagnostic criteria of schizophrenia, schizotypal and delusional disorders according to ICD-10 and signed informed consent forms as approved by the local ethics committee. They did not suffer from any other axis I disorder, including substance abuse. Diagnoses were confirmed using chart information and reports from clinicians who had treated these patients. Patients who had previously been receiving antipsychotic medication underwent a washout period of 3–5 days. Antipsychotics were chosen by the psychiatrists treating the patients, dosing followed clinical needs within the recommended labeled dose ranges.

At baseline as well as after 2, 4, and 12 weeks of treatment, psychopathology was rated by means of the Positive and Negative Syndrome Scale (PANSS) [29]. At the same points in time, the severity of illness and overall level of functioning were assessed using the Global Assessment of Functioning Scale (GAF) [30, 31]. Additionally, side effects were quantified using the Udvalg for Kliniske Undersogelser (UKU) Side Effect Rating Scale [32] at weeks 2, 4, and 12. The UKU comprises a total of 48 symptoms, arranged into four groups: psychic, neurological, autonomic and other side effects. Each symptom is scored on a severity scale from 0 to 3, and the rater assesses whether the report is best attributed to a side effect (rated as improbable, possible or probable) or related to the disease. Only adverse effects with scores ≥1 on any UKU item and a causal relationship of possible or probable, were considered as antipsychotic induced side effects.

After 2, 4, and 12 weeks of treatment, the patients’ subjective well-being as well as their subjective response to and their attitudes towards medication were assessed by means of the short form of the SWN (SWN-K) and the DAI, respectively.

The SWN-K represents a self-rating Likert scale with 6 response categories (1 = not at all, 6 = very much) [20]. It consists of 20 statements (10 positive and 10 negative) on 5 subscales (“mental functioning”, “emotional regulation”, “social integration”, “physical functioning”, “self-control”) with a minimum total score of 20 and a maximum total score of 120; higher scores indicate better well-being. In accordance with the guidelines, scoring of negatively worded items was reversed (1 → 6, 2 → 5, …, 6 → 1) before total score and subscores were calculated [20]. The criterion for adequate subjective well-being is met if an SWN-K total score ≥ 80 is achieved [33, 34].

The DAI, on the other hand, is a 30-item self-report questionnaire consisting of statements about perceived effects and benefits of antipsychotics with which the patient can agree or disagree. It is divided into 7 factors: (1) subjective positive feelings related to antipsychotics (e.g., feeling happier), (2) subjective negative feelings attributed to the drugs (e.g., feeling tired and sluggish), (3) health/illness-dependent drug intake: patients’ model of health (e.g., believing it is unnatural to take medication), (4) patients’ confidence in the physician (e.g., believing it is up to the doctor when one stops taking medication), (5) control: patients’ attitudes toward the locus of control in taking medication (e.g, feeling pressured to ingest medication), (6) prevention: patients’ belief in the effect of antipsychotics in forestalling relapse (e.g., antipsychotics can prevent getting sick), and (7) harm: patients’ concerns with potential toxic effects (e.g., believing medication is a slow acting poison). Each item oft the DAI is scored 1 or 2, depending on whether the answer selected by the patient indicates a negative or positive view of medication. Raw scores of the DAI (total score and subscales) were linearly transformed to a common range from 0 to 100 to facilitate interpretation [35]. For the total score and for those DAI subscales addressing positive aspects, higher scores reflect a more positive drug attitude. For DAI subscales dealing with negative aspects higher scores indicate a more negative drug attitude.

### Statistical methods and power analysis

Prior to the analysis all metric variables were checked for deviations from normality by investigating their skewness, G_1._ Values of G_1_ above 0.5 or below − 0.5 were regarded as sizable deviation from normality indicating the need for non-parametric testing. Changes of PANSS, GAF, SWN-K and DAI over the course of time were analyzed by repeated-measures ANOVA or by Friedman test and subsequent Wilcoxon matched-pairs tests, depending on the variable type (normally or non-normally distributed, respectively). Missing observations were omitted listwise in the repeated-measures analyses (i.e., if one time point was missing the patient as a whole was disregarded in the respective analysis). However, missing values were very rare as all patients completed the SWN-K and 29 of 30 (96.7%) completed the DAI at all planned assessment times (week 2, 4 and 12). Associations of SWN-K and DAI scores with patient characteristics, symptomatology (PANSS), side effects (UKU) and GAF were investigated by means of the Spearman rank correlation coefficient, as part of the variables involved were non-normally distributed.

The sample size of *n* = 30 patients provides sufficient power (1-β = 0.8) to detect in a repeated-measures ANOVA under standard assumptions (type-one error α= 0.05; correlations among repeated measures, *r* = 0.5; non-sphericity correction, ε = 0.9) effect sizes of f = 0.236 for the comparison of 4 assessments and f = 0.258 for comparing 3 assessments. These are medium effect sizes according to Cohen’s classification [36]. Moreover, the sample size of *n* = 30 is sufficient to detect with a power of 1-β=0.8 and a two-tailed type-one-error of 0.05, Spearman rank correlation coefficients r ≥ 0.49 a in a non-parametric correlation analysis. This is a medium to large correlation according to Cohen’s classification (close to the threshold of 0.5 indicating large correlations).

## Results

Demographic and clinical characteristics of the study sample are summarized in Table 1. Data of 30 patients were available for analysis at baseline, week 2 and 4. At week 12, the DAI evaluation was missing for one subject, and the GAF evaluation for another one. None of the patients withdrew within the 12 weeks observation period. They had a mean age of 37.5 ± 9.7 years and a mean duration of illness of 8.7 ± 6.7 years with a range from 2 to 21 years. 57% were male. At baseline, the mean PANSS total score was 59.7 ± 16.5, indicating mild symptom severity. The mean baseline GAF score was 56.7 ± 16.4, thereby indicating a moderate impairment. At baseline, 40% of study participants were inpatients, while the others were treated at a specialized outpatient clinic. All patients started monotherapy with a new-generation antipsychotic drug (amisulpride: *n* = 7, aripiprazole: n = 7, clozapine: *n* = 6, olanzapine: *n* = 3, quetiapine: n = 3, ziprasidone: *n* = 2, sertindole: n = 2). The mean doses and ranges of antipsychotic medication were as follows: amisulpride 428.6 ± 138 mg (200–600 mg), aripiprazole 19.3 ± 15.5 mg (15–30 mg), clozapine 366.7 ± 75.3 mg (300–500 mg), olanzapine 16.7 ± 5.8 mg (10–20 mg), quetiapine 700 ± 173.2 mg (600-900 mg), ziprasidone 140 ± 28.3 mg (120–160 mg), and sertindole 16 ± 0 mg.

**Table 1:** Demographic and Clinical Patient Characteristics (*n* = 30)

Age, mean ± SD, years	37.5 ± 9.7
Sex, n (%), female/male	13(43.3)/17(56.7)
Duration of illness, mean ± SD, years PANSS score at baseline, mean ± SD	8.7 ± 6.7
Total score	59.7 ± 16.5
Positive symptoms	12.1 ± 5.1
Negative symptoms	17.0 ± 7.1
General psychopathology	30.6 ± 8.1
GAF score at baseline, mean ± SD	56.7 ± 16.4

PANSS = Positive and Negative Syndrome Scale; GAF = Global Assessment of Functioning Scale

The time course of disease severity as indicated by PANSS scores, subjective well-being (SWN-K total score), and attitudes toward medication (DAI total score) is depicted in Table 2. The PANSS total score improved significantly from baseline to weeks 4 and 12, respectively. There was no significant improvement from baseline to week 2. Both the PANSS positive and general psychopathology subscores improved from baseline to weeks 2, 4, and 12, whereas the PANSS negative subscore remained virtually unchanged. The GAF score improved from baseline to weeks 4 and 12. At any assessment, SWN-K and DAI scores reflected both adequate subjective well-being and positive attitudes toward antipsychotic treatment, respectively. Neither SWN-K nor DAI total scores changed significantly over the course of time.

**Table 2:** Time course of disease severity, global functioning, subjective wellbeing, and attitudes toward antipsychotic medication

			Observation time						Comparison (Repeated measures ANOVA)				
	Baseline (0)		Week 2 (1)		Week 4 (2)		Week 12 (3)		Overall		(0) vs. (1)	(0) vs. (2)	(0) vs. (3)
	Mean	SD	Mean	SD	Mean	SD	Mean	SD	F	*p*-value	p-value	p-value	p-value
PANSS total score	59.71	16.51	56.40	15.36	53.07**↓**	15.90	52.33**↓**	14.41	6.10	0.001	0.078	0.003	0.001
PANSS positive	12.13	5.14	10.63**↓**	3.83	9.80**↓**	3.34	9.53**↓**^**#**^	3.07	9.02	< 0.001	0.015	0.001	0.001
PANSS negative	17.00	7.07	17.03	6.87	16.30	7.09	16.17	7.47	0.53	0.629	0.964	0.493	0.333
PANSS general	30.58	8.12	28.74**↓**	7.36	26.97**↓**	7.11	26.63**↓**^**#**^	6.15	7.81	< 0.001	0.042	0.001	0.001
GAF	56.67	16.40	59.03	15.34	62.43**↑**^x^	16.46	61.52**↑**	17.36	5.80	0.004	0.078	0.004	0.011
SWN-K total	- ^a^	- ^a^	83.45	18.63	85.91	17.92	87.56	17.14	1.47	0.253	- ^a^	0.984^b^	0.512^b^
SWN-K mental	- ^a^	- ^a^	16.47	5.19	17.14	4.41	17.43	3.87	1.85	0.173	- ^a^	0.190^b^	0.121^b^
SWN-K self-control	- ^a^	- ^a^	15.03	3.61	16.08	3.89	16.00	4.35	1.94	0.156	- ^a^	0.072^b^	0.139^b^
SWN-K emotional	- ^a^	- ^a^	17.63	5.08	17.93	4.80	18.53	4.51	1.58	0.217	- ^a^	0.621^b^	0.088 ^b^
SWN-K physical	- ^a^	- ^a^	17.47	4.70	17.23	4.45	17.94	4.51	0.70	0.476	- ^a^	0.744^b^	0.470^b^
SWN-K spcial	- ^a^	- ^a^	16.87	4.68	17.53	4.49	17.69	4.25	1.31	0.276	- ^a^	0.115^b^	0.209 ^b^
DAI total score	- ^a^	- ^a^	79.93	16.31	81.83	15.88	79.93	16.34	0.29	0.747	- ^a^	0.528^b^	0.999 ^b^
DAI positive	- ^a^	- ^a^	70.83	29.60	71.13	33.49	71.67	33.79	0.02	0.975	- ^a^	0.937 ^b^	0.844 ^b^
DAI negative	- ^a^	- ^a^	18.33	31.36	15.56	26.60	21.39	28.17	0.94	0.397	- ^a^	0.524 ^b^	0.499 ^b^
DAI illness-related	- ^a^	- ^a^	33.89	35.15	27.78	34.00	23.33**↓**	32.93	3.00	0.061	- ^a^	0.118 ^b^	0.032 ^b^
DAI doctor	- ^a^	- ^a^	93.33	21.71	91.67	23.06	91.38	23.41	0.49	0.570	- ^a^	0.326 ^b^	0.330 ^b^
DAI control	- ^a^	- ^a^	8.33	26.53	6.67	25.37	11.67	28.42	2.45	0.110	- ^a^	0.326 ^b^	0.161 ^b^
DAI prevention	- ^a^	- ^a^	91.67	26.53	90.00	30.51	93.10	25.79	0.59	0.489	- ^a^	0.663 ^b^	0.343 ^b^
DAI harm	- ^a^	- ^a^	28.33	38.69	25.00	36.55	28.33	38.69	0.44	0.649	- ^a^	0.423^b^	0.999 ^b^

PANSS = Positive and Negative Syndrome Scale; GAF = Global Assessment of Functioning Scale; SWN-K = Subjective Well-being under Neuroleptic Treatment Scale, short form, DAI = Drug Attitude Inventory

**↓** Significantly lower than at baseline

**↑** Significantly higher than at baseline

^#^Significantly lower than at week 2

^x^Significantly higher than at week 2

^a^Not assessed at day 0

^b^Comparison with day 14

Correlations between SWN-K and DAI and the interrelations between these scales and both symptom level (PANSS) and functioning (GAF) are depicted in Table 3. There was no correlation between SWN-K and DAI total scores at any time point. A significant negative correlation was found between the SWN-K total score and both the total score and the general psychopathology subscore of the PANSS at weeks 2 and 4, but not at week 12. Moreover, the SWN-K total score correlated negatively with the PANSS positive subscore at week 4, whereas no significant correlation was found between the SWN-K total score and the PANSS negative subscore. The DAI total score did neither correlate with the PANSS total score or any of its subscales nor with the GAF score at any point in time. In contrast, the SWN-K total score correlated positively with the GAF score at all time points.

**Table 3:** Correlation of subjective well-being and drug attitude with symptomatology and global functioning. Spearman rank correlation

			PANSS positive	PANSS negative	PANSS general	PANSS total	GAF	DAI total
Week 2	SWN-K total	r Spearman (p-value)	−.274 (.143)	−.323 (.082)	−.419^*^ (.021)	−.375^*^ (.041)	.559^**^ (.001)	.196 (.299)
	DAI total	r Spearman (p-value)	−.017 (.93)	−.095 (.616)	−.206 (.276)	−.197 (.297)	−.006 (.976)	-
Week 4	SWN-K total	r Spearman (p-value)	−.415^*^ (.022)	−.235 (.210)	−.441^*^ (.015)	−.480^**^ (.007)	.642^**^ (.000)	.128 (.5)
	DAI total	r Spearman (p-value)	.084 (.66)	.161 (.394)	.09 (.637)	.19 (.314)	−.203 (.283)	-
Week 12	SWN-K total	r Spearman (p-value)	−.238 (.205)	−.238 (.206)	−.324 (.081)	−.318 (.087)	.576** (.001)^a^	.068 (.727)^a^
	DAI total	r Spearman (p-value)	−.069 (.721)^a^	−.102 (.598)^a^	−.095 (.625)^a^	−.114 (.555)^a^	−.095 (.63)^b^	-

SWN-K = Subjective Well-being under Neuroleptic Treatment Scale, short form; PANSS = Positive and Negative Syndrome Scale; DAI = Drug Attitude Inventory

^a^*N* = 29

^b^*N* = 28

Regarding correlations of patient characteristics, i.e. age, gender, duration of illness, and inpatient vs. outpatient, with SWN-K and DAI scores, no significant correlation was found. Side effects that were most frequently reported within the observation period of 12 weeks (found in ≥10% of patients) were as follows: increased salivation (18.3%), concentration difficulties (15.8%), asthenia (15.8%), weight gain (15%), tension/ inner unrest (15%), hypokinesia (12.5%), failing memory (11.7%), akathisia (10%), and diminished sexual desire (10%). The item tension/ inner unrest was negatively correlated with the SWN-K total score at weeks 2 (*r* = −.362, *p* = .05) and 12 (*r* = −.454, *p* = .012), and with the DAI total score at week 2 (*r* = −.377, *p* = .04). Additionally, the side effect failing memory was negatively correlated with the SWN-K total score at week 12 (*r* = −.367, *p* = .046). No further correlation between side effects and both subjective well-being and drug attitude was found.

## Discussion

The aim of the current study was to assess a potential association between subjective well-being and attitudes toward antipsychotic medication as well as the interrelations between these issues and both symptom severity and functioning in chronic schizophrenia patients starting treatment with a new-generation antipsychotic drug. As expected, both symptoms and global functioning improved over the course of antipsychotic treatment. Most previous studies reported on an improvement of patients‘ subjective well-being over the course of treatment [37–39]. In our sample, however, the SWN-K total score did not change over time. Next to a limited sample size this seemingly contradictive finding could be a result of patient selection, since we investigated chronically ill patients experiencing mild baseline symptomatology and a generally positive attitude toward medication as indicated by high mean DAI total scores. Of note, four distinct clusters of subjective well-being have been described in individuals with schizophrenia: 16% of “patients moderate” “stable high”, 31% “stable moderate”, 33% “stable low”, and merely 20% improving “subjective well-being over the course of treatment” [40]. According to this classification, the patients included into the current study may be seen to have had a stable high subjective well-being, since they consistently achieved a mean SWN-K total score of ˃80. Following Larsen and Gerlach, we hypothesize that the relatively long mean duration of illness of approximately 9 years may have enabled them to accept their illness [41]. In line with previous studies, we did not find consistent correlations between the PANSS or its subscales and the SWN-K total score [23].

The negative correlation between the SWN-K total score and both the PANSS total score and the general psychopathology subscore is in line with the literature [42, 43], and can be explained by the fact that they contain items that include symptoms of anxiety and depression, which may be more critical in influencing subjective well-being rating than any other symptom of schizophrenia.

Our finding of a significant correlation of global functioning with patients’ subjective well-being corroborates the results of previous studies [44, 45]. Accordingly, subjective well-being may directly impact upon a patient’s social, psychological, and occupational functioning and vice versa.

Similar to the SWN-K total score and in line with a previous report [46], the DAI total score did not change over time. Moreover, drug attitude did not correlate with symptom change. Generally, drug adherence has been reported to be predicted by the patients’ subjective responses to and attitudes toward antipsychotics [14]. The high mean DAI total score in our sample reflects a general positive attitude toward antipsychotic medication and underscores once more, that we investigated a selected patient sample. It clearly has to be noted that adherence is generally higher among study samples than in routine care and that our findings are therefore not attributable to all individuals suffering from schizophrenia [1].

Notably and in contrast to our finding of a consistent association between patients’ subjective well-being and functioning, the DAI total score did not correlate with the GAF score at any time point, which corroborates previous findings [47]. However, in contrast to previous reports we did not find a correlation between the SWN-K and DAI total scores [48, 49]. Next to differences in the patient samples studied, this contradictory result can be explained by the scales‘ varying scopes. The DAIs‘ strength lies in measuring compliance and attitudes towards antipsychotic treatment, whereas the SWN-K can be seen as an alternative for a quality of life instrument in patients using antipsychotic medication [48]. One previous study [49] examined a very heterogeneous sample encompassing patients receiving first- and new-generation antipsychotics in different formulations (oral versus long-acting injectable drugs) with 60% of patients having been on the current medication for at least one year. Furthermore, symptom severity has not been reported in this study. Another longitudinal study [48] investigated patients experiencing more severe symptoms at baseline than our sample. Accordingly, these studies are not entirely comparable with ours.

Regarding the impact of antipsychotic-induced side effects on subjective well-being and drug attitude, inconsistent correlations have been found for tension/ inner unrest and failing memory. This finding is supported by previous research that found that the psychiatrists’ beliefs about the tolerance of a particular antipsychotic drug do not necessarily reflect patients’ well being and attitude toward medication [50]. \Additionally, this effect can be interpreted as the intense efforts in both inpatient units and specialized outpatient clinic to actively elicit adverse events and to respond to patients’ concerns quickly.

When interpreting our data, one has to consider a number of limitations. First of all, the sample size was rather small and early noncompliers, who usually stop attending mental health services, have not been included. Furthermore, the fact that only patients with a generally good attitude toward medication took part in the study limits the generalizability of our findings. Lastly, we did not thoroughly assess the reason for switching antipsychotic medication in patients who had previously been receiving antipsychotic drugs.

## Conclusions

In summary, our findings emphasize that, particularly in chronic schizophrenia patients experiencing mild symptoms, the differences between the SWN and DAI may give good reason for the use of both instruments during antipsychotic treatment in clinical practice and/ or research to systematically monitor and adjust treatment with antipsychotic medication. In so doing it may help finding the optimal dosage and type of antipsychotic medication for an individual patient and therefore enhance adherence.

## Abbreviations

DAI: Drug Attitude Inventory
GAF: Global Assessment of Functioning Scale
PANSS: Positive and Negative Syndrome Scale
SWN: Subjective Well-being under Neuroleptic Treatment Scale
SWN-K: Subjective Well-being under Neuroleptic Treatment Scale, short form
